# Systemic Inflammation Response Index Is Associated with the Presence and Extent of Late Gadolinium Enhancement in Acute Myocarditis

**DOI:** 10.3390/jcm15124505

**Published:** 2026-06-10

**Authors:** Meltem Altınsoy, Esin Kurtuluş Öztürk, Nazlı Turan Şerifler, Tuğba Kayhan Altuner, Funda Başyiğit

**Affiliations:** 1 Department of Cardiology, Etlik City Hospital, Ankara 06170, Turkey; nazlituran113@gmail.com (N.T.Ş.); drtugba_kayhan@yahoo.com (T.K.A.); ftuna02@yahoo.com (F.B.); 2 Department of Radiology, Etlik City Hospital, Ankara 06170, Turkey; e.kurtulus@hotmail.com

**Keywords:** acute myocarditis, cardiac magnetic resonance, SIRI, late gadolinium enhancement, inflammation

## Abstract

**Background**: Acute myocarditis is characterized by heterogeneous inflammatory myocardial involvement, and cardiac magnetic resonance (CMR)-derived late gadolinium enhancement (LGE) is an important marker of myocardial injury. However, the relationship between systemic inflammatory burden and quantitative CMR-defined myocardial involvement remains insufficiently characterized. We aimed to evaluate the association between inflammatory indices, particularly the systemic inflammation response index (SIRI), and the presence and extent of LGE in patients with acute myocarditis. **Methods**: This retrospective single-center observational cohort study included clinically suspected acute myocarditis patients who underwent CMR within 4 weeks of symptom onset. Patients were classified according to the presence or absence of LGE on CMR. Inflammatory indices, including the SIRI, systemic immune–inflammation index (SII), multi-inflammatory indices (MII-I and MII-II), and stress hyperglycemia ratio (SHR), were calculated from admission laboratory parameters. Receiver operating characteristic (ROC) analysis, logistic regression, linear regression, and hurdle Poisson regression analyses were performed. **Results**: LGE was present in 62 patients (36.5%). Patients with LGE demonstrated significantly higher inflammatory markers and inflammatory index values. Among all evaluated biomarkers, the SIRI demonstrated the highest discriminatory performance for LGE detection (AUC: 0.944, 95% CI: 0.897–0.973; cut-off > 1.33; sensitivity 93.5%; specificity 85.2%; *p* < 0.001). In multivariable logistic regression analysis, the SIRI remained independently associated with LGE presence (OR: 4.538, 95% CI: 2.570–8.011; *p* < 0.001). The SIRI was also independently associated with both log-transformed LGE percentage and the number of involved myocardial segments. **Conclusions**: Higher SIRI levels were associated with both the presence and extent of CMR-defined myocardial involvement in patients with acute myocarditis and may reflect the burden of early inflammatory myocardial injury. However, these findings should be interpreted cautiously given the retrospective single-center design and require confirmation in larger prospective multicenter studies before potential clinical application.

## 1. Introduction

Myocarditis is an inflammatory disease of the myocardium caused by infectious agents, drugs, toxins, or autoimmune mechanisms. The clinical course of acute myocarditis is highly heterogeneous and ranges from mild self-limited disease to cardiogenic shock, malignant arrhythmias, and sudden cardiac death, depending on the underlying etiology and severity of myocardial involvement [1]. Patients frequently present with non-specific symptoms such as chest pain, dyspnea, and palpitations, often mimicking more common cardiovascular conditions including acute coronary syndromes. In some patients, only transient troponin elevation or electrocardiographic abnormalities may be present following viral illness, vaccination, toxic exposure, or immune-mediated inflammatory activation.

The pathophysiology of acute myocarditis involves a complex interaction between direct myocardial injury and immune-mediated inflammatory responses. Viral infection, autoantibodies, toxins, or other inflammatory triggers may induce cardiomyocyte necrosis and extracellular matrix degradation, subsequently activating innate and adaptive immune pathways [2]. Although inflammatory activation is necessary for clearance of injured tissue, excessive or dysregulated immune responses may contribute to persistent myocardial injury, ventricular remodeling, fibrosis, and heart failure progression. Despite these mechanisms, clinically accessible biomarkers capable of reliably reflecting the degree of myocardial inflammatory involvement remain limited.

Although endomyocardial biopsy (EMB) remains the definitive diagnostic standard for myocarditis, its invasive nature restricts routine clinical application to selected high-risk cases [3]. Consequently, cardiac magnetic resonance (CMR) has become the principal non-invasive imaging modality for tissue characterization in suspected myocarditis. CMR enables comprehensive assessment of myocardial edema, hyperemia, necrosis, and fibrosis through multiparametric imaging sequences incorporated into the updated Lake Louise Criteria [4]. During the acute phase, late gadolinium enhancement (LGE) primarily reflects increased extracellular distribution volume related to myocardial edema, inflammatory injury, and altered sarcolemmal permeability, whereas in later stages it may represent irreversible myocardial damage and replacement fibrosis. Importantly, both the presence and extent of LGE have been associated with adverse cardiovascular outcomes and may provide important prognostic information in the acute myocarditis [5,6]. Furthermore, CMR demonstrates particularly high diagnostic sensitivity during the acute inflammatory phase of myocarditis [7].

Systemic inflammatory activation plays a central role in the development and progression of myocardial injury in myocarditis. Neutrophils, monocytes, and platelets contribute to cytokine-mediated inflammation, oxidative stress, endothelial dysfunction, and microvascular impairment, whereas relative lymphopenia may reflect impaired immune regulation. Composite inflammatory indices—including the systemic immune–inflammation index (SII), systemic inflammatory response index (SIRI), and multi-inflammatory indices (MII-I and MII-II)—integrate these cellular components into a single measure of systemic inflammatory burden. In parallel, the stress hyperglycemia ratio (SHR) reflects acute metabolic stress closely linked to inflammatory signaling pathways.

Given the close mechanistic relationship between systemic inflammation and myocardial injury, inflammatory indices obtained at hospital admission may reflect the extent of myocardial inflammatory involvement and correlate with CMR-defined tissue injury during the acute phase of myocarditis. However, evidence regarding the relationship between composite inflammatory indices and quantitative CMR-defined myocardial involvement remains limited. In particular, the association between the SIRI and both the presence and extent of LGE has not been adequately characterized. The specific scientific gap addressed by the present study is whether systemic inflammatory response indices can serve as a reliable bridge between systemic inflammation and organ-specific myocardial tissue injury in the acute setting, thereby potentially supporting biomarker-guided prioritization for advanced imaging. Therefore, the present study aimed to evaluate the relationship between inflammatory indices—particularly the SIRI—and CMR-defined myocardial involvement in patients with acute myocarditis.

## 2. Methods

**Figure 1:**
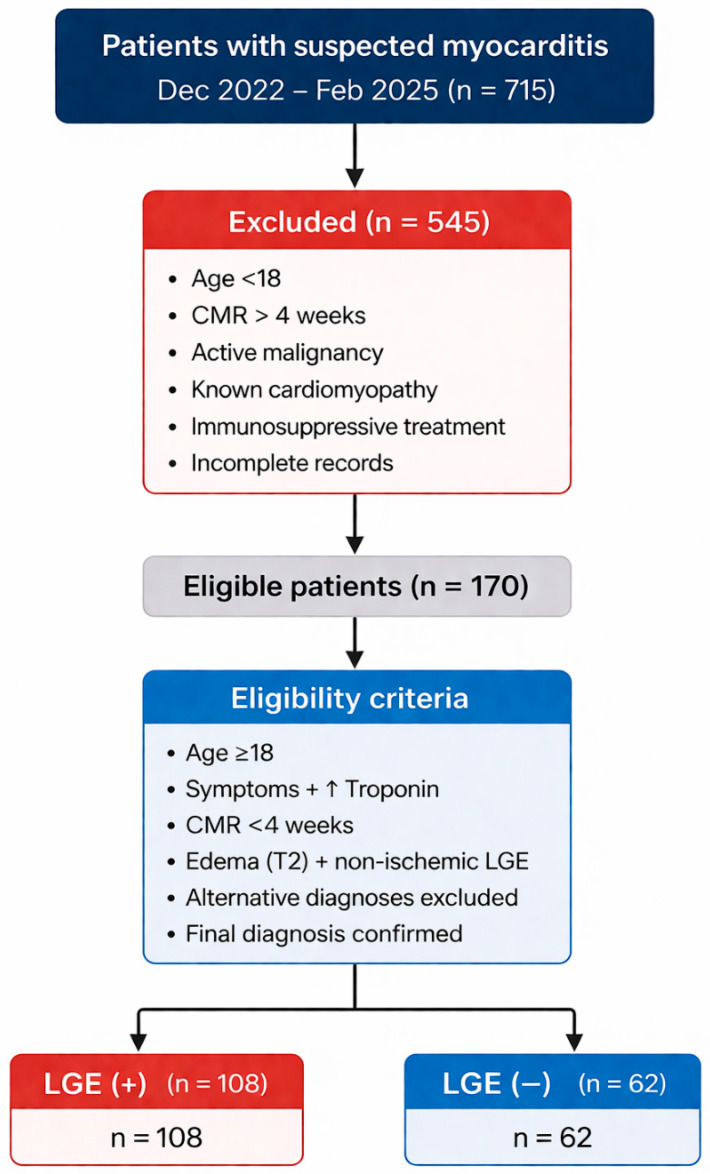
Flowchart of the study.

The population of this retrospective study was recruited from 1 December 2022 to 1 February 2025. Seven hundred and fifteen patients who were ≥18 years old and suspected of acute myocarditis—defined by compatible clinical presentation (e.g., chest pain, dyspnea, or arrhythmia) together with elevated cardiac biomarkers (high-sensitivity troponin)—were identified (Figure 1). One hundred and seventy of these patients, who underwent cardiac magnetic resonance (CMR) imaging within 4 weeks of symptom onset (the time period corresponding to the acute phase according to the 2025 ESC Guideline for the management of myocarditis and pericarditis), were included in the final study population.

The diagnosis of myocarditis was established according to the European Society of Cardiology (ESC) criteria and supported by CMR findings consistent with myocardial involvement, including myocardial edema and/or non-ischemic late gadolinium enhancement (LGE). The study population was divided into two groups based on the presence or absence of LGE in their hearts. Patients with LGE on CMR formed the patient group, while patients without LGE on CMR formed the control group.

Obstructive coronary artery disease was ruled out by either invasive cardiac catheterization or coronary computed tomography prior to CMR in all patients. Exclusion criteria for the study were contraindications for CMR, solid and blood cell cancers, inflammatory and autoimmune diseases, previous acute myocardial conditions (myocarditis or myocardial infarction), or other medical history of cardiac diseases and absence of follow-up troponin since it is determined to be the subacute phase of myocarditis MR scan more than 4 weeks after onset of symptoms [8]. Patients with incomplete records were excluded when essential laboratory measurements, electrocardiographic parameters, or CMR datasets required for study analyses were unavailable.

At our center, patients are diagnosed with myocarditis according to the definition in the ESC myocarditis position paper [9]. We followed the presence of at least one clinical presentation (e.g., acute chest pain, new-onset or worsening dyspnea/heart failure, palpitations/arrhythmias, syncope, aborted sudden cardiac death, or unexplained cardiogenic shock) together with at least one diagnostic criterion from non-invasive testing, including electrocardiographic abnormalities (atrioventricular block, bundle branch block, ST/T wave changes, sinus arrest, or ventricular arrhythmias) and elevated cardiac biomarkers (particularly troponin) or echocardiographic evidence of ventricular dysfunction, in the absence of alternative causes, mostly obstructive coronary artery disease. The ‘COVID-19 vaccine’ variable in our dataset represents the historical vaccination status of the patients. Notably, there were no cases of acute vaccine-associated myocarditis in our study, as no patient had received a COVID-19 vaccine dose within the immediate weeks prior to the onset of symptoms.

The characteristics, comorbidities, primary symptoms, and medications of the patients, as well as the laboratory, clinical, and radiological findings; electrocardiographic (ECG) findings; echocardiographic (echo) findings; and cardiac magnetic resonance (CMR) imaging findings were collected from hospital records. Laboratory data were collected prior to any treatment during the initial health center visit on their complaints.

Laboratory indicators: absolute neutrophil count (ANC), absolute lymphocyte counts (ALC), absolute monocyte counts (AMC), platelet counts (PLT), C-reactive protein (CRP), and percentage of immature granulocytes (IG) at the presentation and follow-up serum troponin (median 8 weeks after diagnosis), N-terminal pro-brain natriuretic peptide (NT-proBNP) levels, liver and kidney function enzymes, and electrolytes were collected. The SII, SIRI, MII-I, and MII-II and the stress hyperglycemia values of all patients in the first 6 h of admission to the cardiology clinic were calculated and recorded. The SII was calculated as SII  =  neutrophil count  ×  platelet count/lymphocyte count; the systemic inflammatory response index (SIRI) = neutrophil count  ×  monocyte count/lymphocyte count; multi-inflammatory index-1 (MII-I) = neutrophil–lymphocyte ratio (NLR) × C-reactive protein (CRP); multi-inflammatory index-2 (MII-II) = platelet–lymphocyte ratio (PLR) × CRP; the stress hyperglycemia ratio (SHR) is calculated by dividing the absolute blood glucose by the estimated average glucose obtained from glycosylated hemoglobin (HbA1c): [glucose (mg/dL)/18]/[(1.59 × HbA1c) − 2.59] [10,11,12].

All the patients had serial standard resting 12-lead ECGs (25 mm/s, 10 mm/mV) throughout their hospitalization. The index of cardiac electrophysiological balance (ICEB) was calculated as the ratio of the QT interval to the QRS duration (ICEB = QT/QRS), as previously described. All measurements were obtained from a standard 12-lead electrocardiogram performed at the time of admission. Echocardiographic imaging was conducted in the left lateral decubitus position utilizing parasternal and apical views with a commercially available device (Vivid 7, GE Medical Systems, Horten, Norway; 3.5 MHz phased array transducer). LVEF was obtained from apical 4-chamber, 2-chamber, and parasternal long axis views.

All patients were scanned with a 1.5 T magnetic resonance device (Philips Achieva; Philips Healthcare, Amsterdam, The Netherlands) using phased-array coils. CMR protocols included cine images with steady-state free precession (SSFP)-oriented 2-chamber vertical long-axis view, 4-chamber horizontal long-axis view, 3-chamber view, and short axis; phase-sensitive inversion recovery (PSIR) images for studying early (1–3 min) and late (10–15 min) gadolinium enhancement performed after intravenous administration of gadolinium (0.1–0.2 mmol/kg); and also native and post-contrast T1 mapping and T2 mapping in adequate short axis slices (basal, middle, and apical). Native and post-contrast T1 mapping were obtained using the MOLLI sequence (TR: 2.8 ms, TE: 1.31 ms). T2 maps were acquired with the mGraSE sequence (TR: 698 ms, TE: 9.3 ms). The presence of myocardial LGE was visually evaluated, and its extent was semi-quantitatively reported according to the American Heart Association’s 17-segment model [13]. Myocarditis was diagnosed according to Lake Louise criteria [4]. The reference values for T1 and T2 mapping used in this study were based on the latest literature with native T1 normal reference values (989 ± 42 ms), ECV normal reference values (23 ± 3), and T2 normal reference values (56 ± 3 ms) [14]. LGE was considered present when identified in at least two orthogonal imaging planes. Quantification of LGE extent was performed using manual planimetry of hyperenhanced myocardial areas on short-axis late gadolinium enhancement images, with careful contour adjustment to optimize accuracy and minimize imaging artifacts. All CMR images were independently evaluated by two experienced observers blinded to clinical and laboratory data. To assess interobserver reproducibility, 45 randomly selected patients were independently re-analyzed at separate time points with an interval of at least 1 week. Interobserver reproducibility analysis demonstrated excellent agreement for both LGE percentage and segmental analyses. For LGE percentage measurements, the intraclass correlation coefficient (ICC) for average measures was 0.990 (95% CI: 0.983–0.995). For segmental LGE analysis, the ICC for average measures was 0.996 (95% CI: 0.993–0.998). All reproducibility analyses were statistically significant (all *p* < 0.001), indicating excellent measurement reliability.

### Statistical Analysis

Statistical analyses were performed using IBM SPSS Statistics for Windows, version 22.0 (IBM Corp., Armonk, NY, USA) and R software (version 4.5.2; R Foundation for Statistical Computing, Vienna, Austria) within the RStudio environment (version 2025.09.2). Categorical variables were expressed as frequencies and percentages, and continuous variables were presented as mean ± standard deviation or median (interquartile range), as appropriate. Normality of distribution was assessed using the Shapiro–Wilk test. Comparisons between patients with and without LGE were performed using the χ^2^ test or Fisher’s exact test for categorical variables and the independent samples *t*-test or Mann–Whitney U test for continuous variables, as appropriate. Receiver operating characteristic (ROC) curve analysis was used to evaluate the diagnostic performance of variables for predicting LGE. The area under the curve (AUC) was calculated, and optimal cut-off values were determined using the Youden index. Comparisons between ROC curves were performed using the DeLong test. Univariable and multivariable logistic regression analyses were conducted to identify independent predictors of LGE presence. Model calibration was assessed using the Hosmer–Lemeshow goodness-of-fit test and graphical calibration assessment. Internal validation of the multivariable logistic regression model was performed using bootstrap resampling with 1000 repetitions, and model stability was assessed using the bootstrap distribution of the area under the ROC curve. Linear regression analysis was performed to evaluate the association between clinical variables and LGE percentage. Variables included in multivariable models were selected based on clinical relevance and univariable analysis (*p* < 0.10). To minimize the risk of overfitting, the number of variables included in multivariable models was limited in relation to the number of outcome events. Multicollinearity was assessed using variance inflation factors (VIF), and no significant collinearity was detected. Variables demonstrating substantial right-skewed distributions, including hs-troponin, NT-proBNP, and CRP, were logarithmically transformed before regression analyses to improve model assumptions and statistical stability. A hurdle Poisson regression model was applied to evaluate the number of LGE-involved myocardial segments, accounting for excess zero counts and separately modeling the presence and extent of myocardial involvement. Spearman correlation analysis was performed to assess the relationship between inflammatory indices (SII, SIRI, MII-I, and MII-II) and both LGE percentage and the number of involved segments. A two-sided *p*-value < 0.05 was considered statistically significant.

Prior to study initiation, ethical approval was obtained from the local ethics committee (decision no. 2024-0052; approval date: 26 February 2025), and the study was conducted in accordance with the principles of the Declaration of Helsinki.

## 3. Results

A total of 170 patients with acute myocarditis were included in the study, of whom 62 (36.5%) demonstrated late gadolinium enhancement (LGE) on CMR, while 108 (63.5%) did not.

Baseline demographic and clinical characteristics were similar between groups (Table 1). There were no significant differences in age, sex distribution, body mass index, or time from symptom onset to CMR (all *p* > 0.05). The prevalence of comorbidities, including hypertension and diabetes mellitus, as well as smoking status and COVID-19 vaccination history and medications, was also comparable between the two groups.

Among presenting symptoms, dyspnea was more frequent in patients with LGE (44.6% vs. 30.3%, *p* = 0.001), whereas arrhythmia (16.1% vs. 26.7%, *p* = 0.011), syncope (6.6% vs. 11.9%, *p* = 0.044), and palpitations (15.7% vs. 25.7%, *p* = 0.019) were more common in patients without LGE. The frequency of recent infection and chest pain did not differ significantly between groups.

Electrocardiographic parameters showed significant differences between groups. Patients with LGE had higher QTc intervals (404.0 vs. 396.0 ms, *p* = 0.024), wider frontal QRS–T angles (36.0 vs. 24.5 degrees, *p* = 0.003), higher Tpe intervals (80.0 vs. 79.0 ms, *p* = 0.039), and elevated ICEB values (4.61 vs. 4.30, *p* = 0.013). QRS duration showed no significant difference.

CMR findings demonstrated that LGE-positive patients had a median of 3 (2–4) involved segments and a median LGE extent of 6% (4–8%), whereas no LGE was observed in the LGE-negative group (both *p* < 0.001).

Left and right ventricular volumetric parameters and systolic function were largely comparable between groups. There were no statistically significant differences in LV EDVi, LV ESVi, LVEF, RV EDVi, RV ESVi, or RVEF (all *p* > 0.05), although borderline differences were observed in LV stroke volume index and LVEF.

Regarding baseline laboratory parameters (Table 2), patients with LGE had higher serum glucose levels compared with those without LGE (107.5 [28.8] vs. 96.0 [20.0] mg/dL, *p* < 0.001), while HbA1c levels were similar between groups (*p* = 0.765). Liver enzymes were significantly higher in the LGE group, including alanine aminotransferase (ALT) (29.0 [22.3] vs. 23.0 [14.8] U/L, *p* = 0.001) and aspartate aminotransferase (AST) (44.5 [59.5] vs. 24.0 [22.0] U/L, *p* < 0.001). Lipid parameters, including LDL-C, HDL-C, and triglyceride levels, did not show significant differences between groups.

Markers of myocardial injury and hemodynamic stress were significantly elevated in patients with LGE. High-sensitivity troponin levels were higher in the LGE group (568.5 [1044.5] vs. 73.0 [309.1], *p* < 0.001), and NT-proBNP levels were also increased (205.0 [478.0] vs. 32.5 [73.0], *p* < 0.001). Follow-up troponin levels remained higher in patients with LGE (22.0 [43.75] vs. 4.0 [13.98] ng/L, *p* < 0.001). Inflammatory burden was greater in the LGE group, as reflected by higher CRP values (53.75 [91.25] vs. 17.5 [56.48] mg/L, *p* < 0.001).

Hematologic parameters demonstrated significant differences in leukocyte subtypes. Neutrophil counts were higher in the LGE group (7.355 [2.758] vs. 4.095 [2.048] × 10^3^/µL, *p* < 0.001), whereas lymphocyte counts were lower (1.980 [1.230] vs. 2.670 [1.200] × 10^3^/µL, *p* < 0.001). Monocyte counts were significantly higher in patients with LGE (1.000 [405] vs. 560 [309] × 10^3^/µL, *p* < 0.001), and immature granulocyte percentage was also increased (0.40 [0.23] vs. 0.30 [0.20], *p* = 0.001). Hemoglobin and platelet counts did not differ significantly between groups.

Inflammatory indices were markedly higher in patients with LGE. SII values were significantly elevated in the LGE group compared with the non-LGE group (892.3 vs. 397.6 [239.4], *p* < 0.001). Similarly, the SIRI (3.82 [3.89] vs. 0.93 [0.54], *p* < 0.001), MII-I (197.4 [401.2] vs. 26.4 [97.1], *p* < 0.001), and MII-II (7946.0 [11,480.2] vs. 1711.0 [4748.6], *p* < 0.001) were all significantly higher in the LGE group. The stress hyperglycemia ratio was also increased in patients with LGE (1.13 [0.29] vs. 1.01 [0.22], *p* = 0.001). Additionally, the index of corrected electrophysiological balance (ICEB) was higher in the LGE group (4.61 [0.87] vs. 4.30 [0.63], *p* = 0.013).

**Figure 2:**
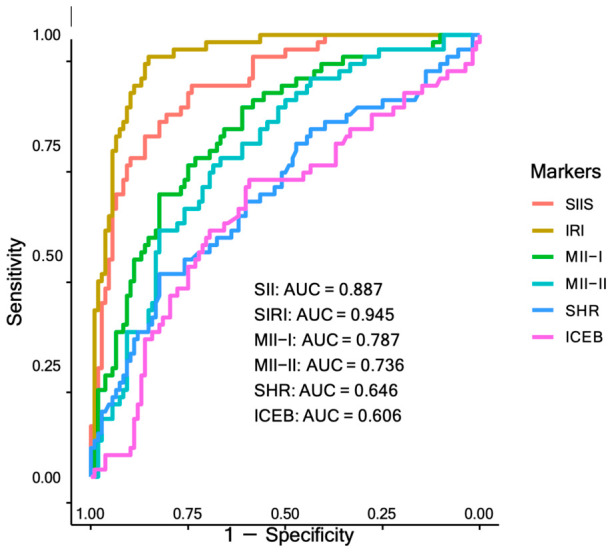
Comparative ROC Analysis for prediction of LGE presence.

As shown in Table 3 and demonstrated in Figure 2, Receiver operating characteristic (ROC) curve analysis was performed to evaluate the diagnostic performance of inflammatory and electrophysiological indices for predicting the presence of late gadolinium enhancement (LGE) in patients with acute myocarditis.

Among the evaluated biomarkers, the SIRI demonstrated the highest discriminatory performance, with an AUC of 0.944 (95% CI 0.897–0.973). Using a cutoff value of >1.33, the SIRI yielded a sensitivity of 93.5% and a specificity of 85.2% (*p* < 0.001). The SII demonstrated strong discriminatory capacity with an AUC of 0.893 (95% CI 0.836–0.935); at a cut-off value of >617.5, the sensitivity and specificity were 77.4% and 87.0%, respectively (*p* < 0.001). MII-I yielded an AUC of 0.791 (95% CI 0.722–0.850), with a cutoff value of >134.31 providing a sensitivity of 62.9% and specificity of 82.4% (*p* < 0.001). MII-II demonstrated an AUC of 0.738 (95% CI 0.666–0.803), with a cut-off value of >3719.76, corresponding to a sensitivity of 69.4% and specificity of 68.5% (*p* < 0.001). The SHR showed modest discrimination with an AUC of 0.648 (95% CI 0.571–0.719). At a cutoff value of >1.19, the SHR demonstrated a sensitivity of 45.2% and a specificity of 82.4% (*p* = 0.001). The ICEB exhibited limited diagnostic performance with an AUC of 0.614 (95% CI 0.536–0.687); a cutoff value of >4.42 yielded a sensitivity of 66.1% and a specificity of 59.3% (*p* = 0.015) (Figure 2). Pairwise comparison of ROC curves using the DeLong test demonstrated that the SIRI had a significantly higher AUC compared with the SII, MII-I, MII-II, SHR, and ICEB (all *p* < 0.01) (Supplement Table S2).

Univariate and multivariate logistic regression analyses were conducted to determine factors associated with the presence of late gadolinium enhancement (LGE) in patients with acute myocarditis (Table 4).

In univariate analysis, the systemic inflammation response index (SIRI) was significantly associated with LGE (OR 5.077, 95% CI 3.015–8.548; *p* < 0.001). The frontal QRS–T angle also showed a significant association with LGE (OR 1.019, 95% CI 1.008–1.031; *p* = 0.001). In addition, high-sensitivity troponin (OR 1.001, 95% CI 1.001–1.002; *p* < 0.001), NT-proBNP (OR 1.003, 95% CI 1.001–1.004; *p* < 0.001), serum glucose (OR 1.027, 95% CI 1.010–1.044; *p* = 0.002), and lnCRP (OR 1.571, 95% CI 1.257–1.962; *p* < 0.001) were also significantly associated with LGE. Conversely, the ICEB and platelet count were not significantly related to LGE. To identify independent predictors of LGE, a multivariate logistic regression model was used.

**Figure 3:**
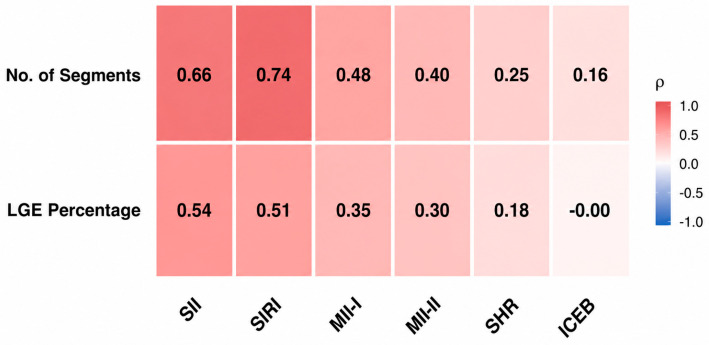
Spearman correlation heatmap demonstrating the association between inflammatory indices and the number of LGE involvement segment and the LGE percentage.

The results indicated that the SIRI remained the only significant independent predictor of LGE (OR: 4.538, 95% CI: 2.570–8.011, *p* < 0.001). After adjusting for confounding factors, the previously significant associations for fQRS-T angle, hs-Troponin, NT-Pro BNP, Glucose, and ln(CRP) were no longer statistically significant (all *p* > 0.05), suggesting that the inflammatory burden reflected by the SIRI has a superior predictive value for LGE (Figure 3). No significant multicollinearity was observed among predictors included in the multivariable model. VIF values were low for all variables (SIRI: 1.29, fQRS-T: 1.05, hs-troponin: 1.29, NT-proBNP: 1.45, glucose: 1.03, and ln(CRP): 1.28) Model validation analyses are presented in Supplementary Table S4. The Hosmer–Lemeshow goodness-of-fit test suggested suboptimal calibration of the multivariable logistic regression model (χ^2^ = 32.84, df = 8, *p* < 0.001). However, bootstrap internal validation with 1000 resamples demonstrated preserved discriminatory performance, with a mean AUC of 0.940 (95% CI 0.891–0.979). Graphical calibration assessment showed generally preserved agreement between predicted probabilities and observed frequencies across risk groups, although some degree of miscalibration was observed in lower predicted probability ranges (Supplementary Figure S2). Additional sensitivity analysis including the interval between symptom onset and CMR acquisition as a continuous covariate is presented in Supplementary Table S5. After adjustment for imaging timing, the SIRI remained independently associated with LGE presence, whereas the interval between symptom onset and CMR acquisition was also independently associated with LGE detection.

The SIRI showed the strongest correlation with both LGE extent and segment involvement, whereas other indices demonstrated weaker or negligible associations (Figure 3). 

Linear regression analysis was performed to identify factors associated with LGE percentage, as shown in Table 5. The SIRI was significantly associated with log-transformed LGE percentage in both univariable and multivariable analyses (β = 0.113, *p* < 0.001), and it was the only independent predictor. Other variables were not significantly associated.

Linear regression analysis was performed to identify factors associated with the number of LGE-involved myocardial segments in LGE-positive patients (Table 6). The SIRI was significantly associated with the number of involved segments in both univariable (β = 0.314, 95% CI 0.192–0.436, *p* < 0.001) and multivariable analyses (β = 0.319, 95% CI 0.164–0.473, *p* < 0.001). No other clinical or laboratory variables, including lnProBNP, the ICEB, age, sex, LVEF, and lnCRP, were significantly associated with segment involvement.

To further evaluate predictors of both the presence and extent of myocardial involvement, a hurdle Poisson regression model was applied (Supplement Table S1). In the zero hurdle (logistic) component, the SIRI was independently associated with LGE presence (OR = 3.20, 95% CI 2.05–5.00, *p* < 0.001), whereas ln hs-troponin, lnProBNP, and the ICEB were not significant predictors.

In the count component of the model, which assessed the number of involved segments among LGE-positive patients, the SIRI remained significantly associated with LGE extent (rate ratio = 1.10, 95% CI 1.05–1.16, *p* < 0.001). No significant associations were observed for ln hs-troponin, lnProBNP, or the ICEB.

Late gadolinium enhancement (LGE) distribution was analyzed according to the American Heart Association’s 17-segment model (Supplement Table S3). LGE was most frequently observed in the mid-inferior segment (segment 10, 45.1%) and the basal inferior lateral segment (segment 5, 40.3%). Other commonly affected regions included the mid-inferior lateral (segment 11, 38.7%) and mid-anterior lateral (segment 12, 22.5%) segments.

## 4. Discussion

Acute myocarditis is a clinically heterogeneous condition characterized by variable degrees of myocardial inflammation and injury. In this study, the SIRI showed a strong and consistent association with both the presence and extent of late gadolinium enhancement (LGE) in patients with acute myocarditis. Patients with LGE exhibited significantly higher levels of systemic inflammatory biomarkers, myocardial injury markers, and selected electrocardiographic repolarization parameters compared with those without LGE. Importantly, cardiac magnetic resonance (CMR) imaging was performed within the first four weeks of presentation. Therefore, LGE in our cohort should be interpreted in the context of early imaging; LGE likely represents predominantly acute myocardial injury and inflammation rather than mature replacement fibrosis, including edema, inflammation, necrosis, and expansion of the extracellular space [15].

The most prominent finding of our analysis is that the SIRI was independently associated with LGE presence, with an approximately fourfold increase in odds (OR 4.538, 95% CI [2.570–8.011]) in multivariable analysis. Given the early timing of imaging, this finding suggests that systemic inflammatory burden may play a central role in the initiation of myocardial involvement rather than chronic fibrotic remodeling. In contrast, biomarkers reflecting myocardial injury (hs-troponin), hemodynamic stress (NT-proBNP), and electrophysiological parameters (ICEB) did not retain significance after adjustment, emphasizing the potential importance of inflammation in the early phase of myocarditis.

Myocarditis is a dynamic, multi-phase process. In the initial phase, infectious or toxic triggers directly injure cardiomyocytes. This is followed by an immune-mediated phase characterized by cytokine release, immune cell infiltration, and activation of both innate and adaptive immune responses. Finally, the disease either resolves or progresses toward chronic inflammation and myocardial fibrosis [16]. In the acute phase, the intensity of systemic inflammation is closely linked to the extent of myocardial injury. In this context, hemogram-derived inflammatory indices have gained increasing attention, as they provide a more integrative representation of immune activation compared with isolated leukocyte counts.

Among non-invasive diagnostic modalities, CMR remains the reference standard for the assessment of myocardial inflammation and tissue characterization, particularly when applying the updated Lake Louise Criteria [4]. In our cohort, patients with LGE demonstrated a distinct inflammatory phenotype characterized by elevated CRP levels, neutrophilia, relative lymphopenia, and increased values of composite inflammatory indices, including the SII, SIRI, MII-I, and MII-II. This pattern is consistent with immune-mediated myocardial injury triggered by innate immune activation and dysregulated inflammatory responses.

In addition to demonstrating strong statistical performance, the identified cut-off values for inflammatory indices—particularly the SIRI—may have important clinical implications. Given the observed association with CMR—defined myocardial involvement, the SIRI may potentially represent a supportive biomarker associated with a higher likelihood of significant acute myocardial inflammation. These findings should be interpreted cautiously and considered hypothesis-generating until validated in larger prospective cohorts with longitudinal outcome assessments. However, because CMR was performed during the early phase of myocarditis in the present study, LGE findings likely reflect acute inflammatory injury, edema, and extracellular expansion rather than irreversible fibrosis. Moreover, the absence of follow-up CMR imaging limits assessment of whether these early LGE findings persist or resolve over time. Therefore, the clinical implications of early LGE and the proposed utility of the SIRI should be interpreted cautiously. However, these thresholds should be interpreted with caution, as they were derived from a single-center cohort and require external validation before implementation in routine clinical practice.

Importantly, comparative analysis of ROC curves using the DeLong test demonstrated that the SIRI had significantly higher discriminatory performance compared with the SII, MII-I, MII-II, SHR, and ICEB. This finding suggests that the SIRI may provide stronger discriminatory performance than several other commonly used inflammatory and electrophysiological indices in the acute phase of myocarditis.

Beyond the presence of LGE, the SIRI was also independently associated with both segment-based and quantitative measures of myocardial involvement, including the number of LGE-involved myocardial segments and LGE percentage. These associations remained consistent across multiple analytical approaches, including linear regression and hurdle Poisson models. Given the early timing of CMR acquisition, these findings suggest that systemic inflammatory burden is associated not only with the presence but also with the spatial extent and magnitude of acute myocardial inflammatory involvement. The use of log transformation for LGE percentage further supported the robustness of these findings by appropriately accounting for skewed data distribution.

An additional important finding is the dissociation between inflammatory markers and classical cardiac biomarkers after adjustment. Although hs-troponin and NT-proBNP were significantly associated with LGE presence in univariable analyses, these associations did not persist in multivariable models and were not correlated with LGE extent. This suggests that biomarkers of cardiomyocyte injury primarily reflect acute cellular damage, whereas imaging-based parameters such as LGE capture tissue-level involvement, including inflammatory infiltration and interstitial expansion. This interpretation is supported by histopathological and imaging studies demonstrating that early gadolinium enhancement reflects membrane disruption, edema, and inflammatory cell infiltration rather than established fibrosis [4,17].

In addition, myocardial tissue characteristics evolve dynamically over time in myocarditis. T2-based edema markers typically decrease during recovery and may normalize within weeks to months, further highlighting the importance of imaging timing in interpreting CMR findings [18]. Therefore, in our cohort—where imaging was performed within the first 4 weeks (median 20 days) after symptom onset—LGE should primarily be interpreted as a marker of acute myocardial involvement rather than chronic scar formation. Accordingly, caution is warranted when extrapolating these findings to long-term prognostic implications. Additional sensitivity analysis incorporating the interval between symptom onset and CMR acquisition into the multivariable model demonstrated that imaging timing itself was independently associated with LGE presence. This finding further supports the concept that acute-phase myocardial edema and membrane permeability dynamically influence LGE detectability during the early inflammatory period. Nevertheless, the SIRI remained independently associated with LGE after adjustment for imaging timing, suggesting that the observed relationship was not solely explained by temporal variability in CMR acquisition.

The strong association between the SIRI and LGE observed in our study reinforces the concept that systemic inflammation is closely linked to myocardial involvement in acute myocarditis. The SIRI integrates neutrophil, monocyte, and lymphocyte counts, thereby reflecting the balance between innate immune activation and adaptive immune regulation. This is particularly relevant in myocarditis, where monocyte–macrophage pathways play a critical role in both myocardial injury and repair processes. Elevated SIRI levels may therefore indicate an intensified inflammatory response characterized by neutrophil-mediated cytotoxicity and increased monocytic infiltration [19]. The conversion of systemic inflammatory activation into quantifiable, organ-specific tissue injury is often governed by a complex interplay between the inflammatory trigger and the patient’s genetic background. In the setting of immune-mediated cardiomyopathies, specific molecular predispositions—particularly those involving desmosomal protein abnormalities—can exacerbate the myocardial response to systemic inflammatory signals. As highlighted by Bartoli et al. [20], such genetic backgrounds can predispose individuals to inflammatory ‘hot phases,’ where systemic activation translates into identifiable pericardial and myocardial involvement. Our findings, demonstrating a robust association between the SIRI and LGE extent, align with this concept of biomarker-guided triage. In patients with a vulnerable molecular background, systemic indices like the SIRI may reflect the intensity of an inflammatory process that leads to significant and non-invasively quantifiable tissue damage on CMR. These findings support the potential complementary role of inflammatory biomarkers alongside CMR-based tissue characterization in identifying patients with a greater likelihood of clinically relevant myocardial involvement [21,22].

Furthermore, dysregulated monocytic responses have been implicated in adverse remodeling and progression toward myocardial fibrosis in myocarditis and inflammatory cardiomyopathies [23]. Although our study focuses on early-phase imaging, these mechanisms may potentially contribute to subsequent structural remodeling; however, long-term imaging follow-up was not available in the present study. Also, direct evidence regarding the prognostic significance of the SIRI specifically in acute myocarditis remains limited; recent studies support the clinical relevance of SIRI-level inflammatory burden in myocarditis severity. Kangel et al. reported that SIRI values were higher in patients with fulminant myocarditis compared with non-fulminant myocarditis, suggesting that the SIRI may reflect a more severe inflammatory phenotype [24]. Similarly, Wang et al. demonstrated that patients with fulminant myocarditis had a significantly higher SIRI and other systemic inflammatory indices than those with mild myocarditis and that these indices correlated with NYHA functional class at admission [25]. In addition, previous studies using related hemogram-derived markers, such as neutrophil-to-lymphocyte and monocyte-to-lymphocyte ratios, have suggested that systemic inflammatory activation may be associated with clinical course in myocarditis. Furthermore, the extent of LGE—which we found to be strongly predicted by the SIRI—is an established independent predictor of major adverse cardiac events (MACE), including sudden cardiac death and heart failure hospitalization [3]. Taken together, these findings support the biological plausibility that a higher SIRI may reflect greater inflammatory disease activity in myocarditis. However, because our study lacks longitudinal clinical outcomes and follow-up CMR, the SIRI should be interpreted as a marker associated with early CMR-defined myocardial involvement rather than a validated prognostic tool.

Although other composite inflammatory indices such as SII and MII variants were also elevated in patients with LGE, the SIRI demonstrated the most robust and consistent association across all statistical models. This suggests that the SIRI may capture key pathophysiological pathways linking systemic inflammation to myocardial involvement more effectively than other indices.

Our findings also demonstrated that hs-troponin and NT-proBNP levels were significantly higher in patients with LGE. However, the lack of independent association after adjustment indicates that these biomarkers may represent downstream effects of inflammation rather than primary drivers of myocardial involvement [26].

Similarly, electrocardiographic parameters differed between groups. Patients with LGE demonstrated prolonged QTc intervals, higher ICEB values, and wider frontal QRS–T angles. Previous studies have shown that these ECG markers are associated with myocardial injury and adverse outcomes in myocarditis [6,27]. However, although the ICEB was associated with LGE presence in univariable analysis, it did not retain significance after multivariable adjustment, suggesting that electrophysiological alterations may be secondary to inflammatory and structural changes. Interestingly, arrhythmia-related symptoms such as palpitations and syncope were more frequently observed in the LGE-negative group, whereas dyspnea predominated in patients with visible LGE involvement. Although LGE is generally considered a marker of arrhythmogenic substrate formation, particularly in chronic fibrotic cardiomyopathies, the pathophysiological mechanisms may differ in acute myocarditis. During the early inflammatory phase, transient myocardial edema, diffuse inflammatory involvement below the spatial resolution of CMR, cytokine-mediated ion channel dysfunction, autonomic imbalance, and temporary conduction abnormalities may provoke rhythm-related symptoms even in the absence of detectable focal enhancement. Conversely, visible LGE in the acute phase may reflect a greater burden of myocardial inflammation and extracellular expansion, potentially contributing more prominently to dyspnea-related symptoms. Experimental and clinical studies have shown that inflammatory cytokines and immune-mediated electrophysiological alterations may contribute to arrhythmogenesis independently of established fibrotic scar formation [2,28]. Taken together, these findings indicate that inflammatory, structural, and electrophysiological alterations in myocarditis are closely interrelated but may not evolve in parallel.

Overall, the present findings support an association between systemic inflammatory burden and early CMR-defined myocardial involvement in acute myocarditis. However, prospective multicenter studies with longitudinal clinical and imaging follow-up are required before these biomarkers can be incorporated into routine clinical decision-making.

### Limitations

Several limitations should be addressed. The relatively small patient population may limit statistical power and generalizability. Second, the early timing of CMR, while providing insight into acute inflammatory processes, does not allow for differentiation between reversible injury and permanent fibrosis; in addition, the absence of serial or follow-up CMR imaging prevented assessment of the temporal evolution, persistence, or resolution of LGE findings over time. The single-center, retrospective nature of our study implies that the SIRI cutoff value of >1.33 identified in our cohort may not be directly applicable to different clinical settings with varying laboratory protocols. Furthermore, the lack of long-term outcome data, such as MACE or persistent ventricular dysfunction, limits our ability to definitively conclude the prognostic value of the SIRI. These findings should be considered hypothesis-generating, highlighting the need for multicenter validation and longitudinal follow-up to establish the SIRI’s place in the clinical management of myocarditis. Another important limitation is the absence of routine endomyocardial biopsy confirmation. Although the diagnosis of acute myocarditis was established using clinical presentation and contemporary CMR criteria in accordance with current guideline-based practice, histopathological confirmation was unavailable in most patients. Therefore, other inflammatory cardiomyopathies (for example, viral, autoimmune, or COVID-19 vaccine-associated or idiopathic myocarditis) or uncommon myocarditis subtypes, including giant cell myocarditis or eosinophilic myocarditis, cannot be completely excluded; accordingly, the relationship between the SIRI and LGE may differ across distinct myocarditis etiologies and inflammatory phenotypes. Since various etiologies can activate divergent immunological pathways and inflammatory profiles, this constitutes a potential source of residual confounding that must be tackled in subsequent studies through systematic etiological characterization. Finally, findings require external validation in independent cohorts.

## 5. Conclusions

The SIRI was independently associated with both the presence and extent of myocardial involvement in patients with acute myocarditis and may reflect the burden of early inflammatory injury detected by CMR. Although these findings support a potential association between systemic inflammatory burden and CMR-defined myocardial involvement in acute myocarditis, the results should be considered hypothesis-generating and require validation in larger prospective multicenter cohorts before routine clinical application.

## Figures and Tables

**Table 1:** Comparison of demographic and clinical characteristics parameters according to the presence of Late Gadolinium Enhancement (LGE) on CMR in patients with acute myocarditis.

	LGE Absent n = 108	LGE Present n = 62	*p* Value
Age, years	26 (21–35)	25 (22–35)	0.567
Gender, male, n (%)	92 (85.2)	57 (91.9)	0.198
BMI (kg/m^2^)	23.6 ± 5.9	22.9 ± 6.8	0.431
Days from onset of symptoms to the cardiac CMR scan	21 (15–28)	20 (16–29)	0.533
LGE extent (number of segment involved)	0	3 (2–4)	<0.001
Percentage of LGE	0	6 (4–8)	<0.001
Hypertension, n (%)	6 (5.6%)	2 (3.2%)	0.478
Diabetes Mellitus, n (%)	5 (4.6%)	2 (3.2%)	0.478
Smoking, n (%)	33 (30.6%)	17 (27.4%)	0.666
COVID-19 vaccine, n (%)	53 (49.1%)	28 (45.2%)	0.623
Symptoms			
Recent Infection, n (%)	26 (24.1%)	20 (32.3%)	0.064
Chest pain, n (%)	18 (17.3%)	12 (18.7%)	0.618
Dyspnea, n (%)	33 (30.3%)	28 (44.6%)	0.001
Arrhythmia, n (%)	29 (26.7%)	10 (16.1%)	0.011
Syncope, n (%)	13 (11.9%)	4(6.6%)	0.044
Palpitations, n (%)	28 (25.7%)	10 (15.7%)	0.019
Medications			
Aspirin, n (%)	45 (41.3%)	26 (42.4%)	0.715
Beta Blockers, n (%)	62 (57.4%)	36 (58.2%)	0.801
ACE-I/ARB, n (%)	48 (44.1%)	28 (45.5%)	0.689
Colchicine, n (%)	102 (95.2%)	60 (96.1%)	0.734
NSAID, n (%)	104 (96.6%)	60 (96.3%)	0.812
Electrocardiography			
QTc (ms)	396.0 (386–410)	404.0 (395–415)	0.024
QRS (ms)	90.0 ± 12.0	88.5 ± 15.0	0.061
fQRS-T (°)	24.5 ± 30.8	36.0 ± 50.8	0.003
Tpe (ms)	79.0 (65–84)	80.0 (70–90)	0.039
ICEB	4.30 ± 0.63	4.61 ± 0.87	0.013
Magnetic Resonance Imaging			
LV EDVi (mL/m^2^)	62.9 ± 19.9	63.9 ± 24.1	0.091
LV ESVi (mL/m^2^)	29.8 ± 21.1	28.7 ± 21.6	0.062
LVEF (%)	65.8 ± 9.5	63.2 ± 7.5	0.077
LV SVi (mL/m^2^)	36.2 ± 8.1	39.9 ± 7.3	0.052
RV EDVi (mL/m^2^)	80.5 ± 24.1	74.4 ± 5.33	0.951
RV ESVi (mL/m^2^)	40.1 ± 15.7	37.70 ± 16.07	0.072
RVEF (%)	50.5 ± 10.4	48.9 ± 9.3	0.470

Abbreviations: ACE-I, Angiotensin Converting Enzyme Inhibitors; ARB, Angiotensin receptor blockers; BMI, Body Mass Index; EDV, End Diastolic Volume; EF, Ejection Fraction; ESV, End Systolic Volume; ICEB, Index of Corrected Electrophysiological Balance; LGE, Late Gadolinium Enhancement; LV, Left Ventricle; CMR, Cardiac Magnetic Resonance; NSAID, Nonsteroidal anti-inflammatory drugs; RV, Right Ventricle; SV, Stroke Volume.

**Table 2:** Comparison of laboratory parameters according to the presence of Late Gadolinium Enhancement (LGE) on CMR in patients with acute myocarditis.

	LGE Absent n = 108	LGE Present n = 62	*p* Value
Glucose (mg/dL)	96.0 (20.0)	107.5 (28.8)	<0.001
HbA1c (%)	5.00 (0.30)	4.95 (0.50)	0.765
Creatine (mg/dL)	0.81 (0.21)	0.82 (0.19)	0.303
Albumin (g/L)	42.0 (4.48)	42.0 (3.0)	0.753
ALT (U/L)	23.0 (14.8)	29.0 (22.3)	0.001
AST (U/L)	24.0 (22.0)	44.5 (59.5)	<0.001
LDL-C (mg/dL)	89.0 (35.5)	89.0 (28.5)	0.783
HDL-C (mg/dL)	40.63 ± 10.45	38.77 ± 12.44	0.322
Triglyceride (mg/dL)	103.5 (77.3)	117.0 (74.3)	0.322
High-Sensitive Troponin	73.0 (309.1)	568.5 (1044.5)	<0.001
NT-proBNP	32.5 (73.0)	205.0 (478.0)	<0.001
Follow-Up Troponin (ng/L)	4.0 (13.98)	22.0 (43.75)	<0.001
CRP (mg/L)	17.5 (56.48)	53.75 (91.25)	<0.001
Hemoglobin (g/dL)	14.8 (1.75)	14.85 (1.55)	0.771
Platelet (×10^3^/µL)	245 (75.5)	250.5 (91.0)	0.584
Neutrophil (×10^3^/µL)	4.095 (2.048)	7.355 (2.758)	<0.001
Lymphocyte (×10^3^/µL)	2.670 (1.200)	1.980 (1.230)	<0.001
Monocyte (×10^3^/µL)	560 (309)	1.000 (405)	<0.001
IG (%)	0.30 (0.20)	0.40 (0.23)	0.001
SII	397.6 (239.4)	892.3	<0.001
SIRI	0.93 (0.54)	3.82 (3.89)	<0.001
MII-I	26.4 (97.1)	197.4 (401.2)	<0.001
MII-II	1711.0 (4748.6)	7946.0 (11,480.2)	<0.001
SHR	1.01 (0.22)	1.13 (0.29)	0.001
ICEB	4.30 (0.63)	4.61 (0.87)	0.013

Abbreviations: ALT, alanine aminotransferase; AST, aspartate aminotransferase; CRP, C-reactive protein; HDL-C, high-density lipoprotein cholesterol; IG, immature granulocyte; ICEB, Index of Corrected Electrophysiological Balance; LDL-C, low-density lipoprotein cholesterol; MII-I, Multi-Inflammatory Index-1; MII-II, Multi-Inflammatory Index-2; NT-proBNP, N-terminal pro-brain natriuretic peptide; SHR, Stress Hyperglycemia Ratio; SII, Systemic Immune–Inflammation Index; SIRI, Systemic Inflammation Response Index.

**Table 3:** Receiver Operating Characteristic (ROC) Analysis of LGE.

	AUC	%95 CI	Cut-Off	Sensitivity (%)	Specificity (%)	*p* Value
SII	0.893	0.836–0.935	>617.5	77.4	87.0	<0.001
SIRI	0.944	0.897–0.973	>1.33	93.5	85.2	<0.001
MII-I	0.791	0.722–0.850	>134.31	62.9	82.4	<0.001
MII-II	0.738	0.666–0.803	>3719.76	69.4	68.5	<0.001
SHR	0.648	0.571–0.719	>1.19	45.2	82.4	0.001
ICEB	0.614	0.536–0.687	>4.42	66.1	59.3	0.015

Abbreviations: AUC, Area Under the Curve; CI, Confidence Interval; ICEB, Corrected Electrophysiological Balance; MII-I, Multi-Inflammatory Index-1; MII-II, Multi-Inflammatory Index-2; SHR, Stress Hyperglycemia Ratio; SII, Systemic Immune–Inflammation Index; SIRI, Systemic Inflammation Response Index.

**Table 4:** Predictors of LGE Presence in Acute Myocarditis.

	Univariate		Multivariate	
	Odds Ratio 95% CI	*p*-Value	Odds Ratio 95% CI	*p*-Value
SIRI	5.077 (3.015–8.548)	<0.001	4.538 (2.570–8.011)	<0.001
ICEB	1.505 (0.865–2.620)	0.148		
fQRS-T Angle	1.019 (1.008–1.031)	0.001	1.008 (0.989–1.027)	0.410
hs-Troponin	1.001 (1.001–1.002)	<0.001	1.000 (0.999–1.001)	0.744
NT-Pro BNP	1.003 (1.001–1.004)	<0.001	1.000 (0.933–1.002)	0.557
Glucose	1.027 (1.010–1.044)	0.002	1.011 (0.988–1.033)	0.354
ln(CRP) *	1.571 (1.257–1.962)	<0.001	0.928 (0.686–1.256)	0.630
Platelet	1.002 (0.997–1.006)	0.513		

Abbreviations: CRP, C-reactive protein; hs-Troponin, high-sensitivity cardiac troponin; ICEB, Index of Corrected Electrophysiological Balance; NT-Pro BNP, N-terminal pro–B-type natriuretic peptide; SIRI, Systemic Inflammation Response Index. * CRP was analyzed after natural logarithmic transformation (lnCRP).

**Table 5:** Linear regression analysis for percentage of LGE extent (log-transformed **).

Variable	Univariable β (95% CI)	*p*-Value	Multivariable β (95% CI)	*p*-Value
SIRI	0.113 (0.069–0.157)	<0.001	0.113 (0.073–0.153)	<0.001
lnCRP	0.111 (−0.007–0.229)	0.065	—	—
ICEB	0.054 (−0.130–0.238)	0.558	—	—
lnTrop	0.010 (−0.082–0.102)	0.825	—	—
lnProBNP	0.056 (−0.031–0.143)	0.206	—	—
Sex	−0.170 (−0.690–0.350)	0.511	—	—

Abbreviations: ICEB: Index of cardiac electrophysiological balance; lnCRP: natural logarithmic transformed C-reactive protein; lnProBNP: natural logarithmic transformed N-terminal pro-B-type natriuretic peptide; lnTrop: natural logarithmic transformed troponin; SIRI: Systemic Inflammation Response Index. ** Percentage of LGE extent was log-transformed to meet linear regression assumptions. Multivariable analysis included variables with *p* < 0.10 in univariable analysis.

**Table 6:** Predictors of LGE extent (number of involved segments) in LGE-positive patients.

Variable	Univariable β (95% CI)	*p*-Value	Multivariable β (95% CI)	*p*-Value
* lnProBNP	0.075 (−0.188–0.338)	0.579	−0.102 (−0.391–0.187)	0.487
SIRI	0.314 (0.192–0.436)	<0.001	0.319 (0.164–0.473)	<0.001
ICEB	0.210 (−0.328–0.748)	0.445	0.122 (−0.358–0.601)	0.616
Age	0.039 (−0.009–0.087)	0.111	0.031 (−0.020–0.082)	0.227
Sex (male)	−0.200 (−1.707–1.307)	0.795	−0.701 (−2.173–0.770)	0.343
LVEF	−0.012 (−0.058–0.034)	0.604	−0.010 (−0.054–0.034)	0.648
** lnCRP	0.235 (−0.116–0.586)	0.195	−0.046 (−0.418–0.326)	0.806

* lnProBNP and ** lnCRP denote variables transformed using the natural logarithm to improve model assumptions. Variables with *p* < 0.10 in univariable analysis were entered into the multivariable model.

## Data Availability

The data that support the findings of this study are available from the corresponding author upon reasonable request.

## Supplementary Materials

The following supporting information can be downloaded at: https://www.mdpi.com/article/10.3390/jcm15124505/s1, Figure S1: Bullseye Plot of LGE Distribution Across Myocardial Segments; Figure S2: Calibration Plot of the Multivariable Logistic Regression Model for Prediction of Late Gadolinium Enhancement; Table S1: Hurdle Poisson Regression Analysis for Predictors of LGE Presence and Extent (number of involved segments); Table S2: Pairwise comparison of ROC curves using the DeLong test; Table S3: Distribution of Late Gadolinium Enhancement Across Myocardial Segments (American Heart Association 17-Segment Model); Table S4: Bootstrap Internal Validation and Calibration Analysis of the Multivariable Logistic Regression Model for LGE Prediction; Table S5: Sensitivity Analysis Including the Interval Between Symptom Onset and CMR Acquisition in the Multivariable Logistic Regression Model for Prediction of LGE Presence.
