# Supplementary material for: Systemic Inflammation Response Index Is Associated with the Presence and Extent of Late Gadolinium Enhancement in Acute Myocarditis
Source: J Clin Med. 2026 Jun 10;15(12):4505. doi: 10.3390/jcm15124505 (PMC13301303; doi:10.3390/jcm15124505)
Supplement: Supplementary file 1 [file jcm-15-04505-s001.zip › jcm-4320469-supplementary.pdf]

| Supplementary Table S1. Hurdle Poisson Regression Analysis for Predictors of LGE Presence and Extent (number of involved segments) | | | | |
|---|---|---|---|---|
| **Variable** | **LGE Presence (Zero Model) OR (95% CI)** | **p-value** | **LGE Extent (Count Model) RR (95% CI)** | **p-value** |
| **SIRI** | **3.20 (2.05 – 5.00)** | **<0.001** | **1.10 (1.05 – 1.16)** | **<0.001** |
| ln hsTrop | 1.07 (0.77 – 1.50) | 0.704 | 1.02 (0.91 – 1.14) | 0.755 |
| lnProBNP | 1.18 (0.84 – 1.66) | 0.358 | 0.98 (0.87 – 1.11) | 0.756 |
| ICEB | 0.92 (0.56 – 1.51) | 0.752 | 1.12 (0.86 – 1.45) | 0.400 |
| Data are presented as odds ratios (OR) for the zero hurdle model (predicting LGE presence) and rate ratios (RR) for the count model (predicting the number of LGE-involved segments among LGE-positive patients), with 95% confidence intervals. | | | | |

| Supplementary Table S2. Pairwise comparison of ROC curves using the DeLong test | | |
|---|---|---|
| **Comparison** | **ΔAUC (95% CI)** | **p-value** |
| SIRI vs SII | 0.057 (0.021–0.094) | 0.002 |
| SIRI vs MII-I | 0.150 (0.085–0.215) | <0.001 |
| SIRI vs MII-II | 0.199 (0.126–0.272) | <0.001 |
| SIRI vs SHR | 0.286 (0.192–0.380) | <0.001 |
| SIRI vs ICEB | 0.339 (0.243–0.434) | <0.001 |

| Supplementary Table S3. Distribution of Late Gadolinium Enhancement Across Myocardial Segments (American Heart Association 17-Segment Model) | | | |
|---|---|---|---|
| **Segment Number** | **Myocardial Segment Location** | **Patients (n)** | **Percentage (%)** |
| **1** | Anterior (Basal) | 1 | 1.6% |
| **2** | Anteroseptal (Basal) | 2 | 3.2% |
| **3** | Inferoseptal (Basal) | 4 | 6.4% |
| **4** | Inferior (Basal) | 15 | 24.1% |
| **5** | Inferolateral (Basal) | 25 | 40.3% |
| **6** | Anterolateral (Basal) | 10 | 16.1% |
| **7** | Anterior (Mid) | 1 | 1.6% |
| **8** | Anteroseptal (Mid) | 7 | 11.2% |
| **9** | Inferoseptal (Mid) | 28 | 45.1% |
| **10** | Inferior (Mid) | 46 | 74.1% |
| **11** | Inferolateral (Mid) | 14 | 22.6% |
| **12** | Anterolateral (Mid) | 2 | 3.2% |
| **13** | Anterior (Apical) | 6 | 9.6% |
| **14** | Septal (Apical) | 0 | 0%** |
| **15** | Inferior (Apical) | 0 | 0%** |
| **16** | Lateral (Apical) | 17 | 27.4% |
| **17** | Apex | 0 | 0%** |

* Based on the American Heart Association 17-segment model.
No LGE was observed in segments 14, 15, and 17.

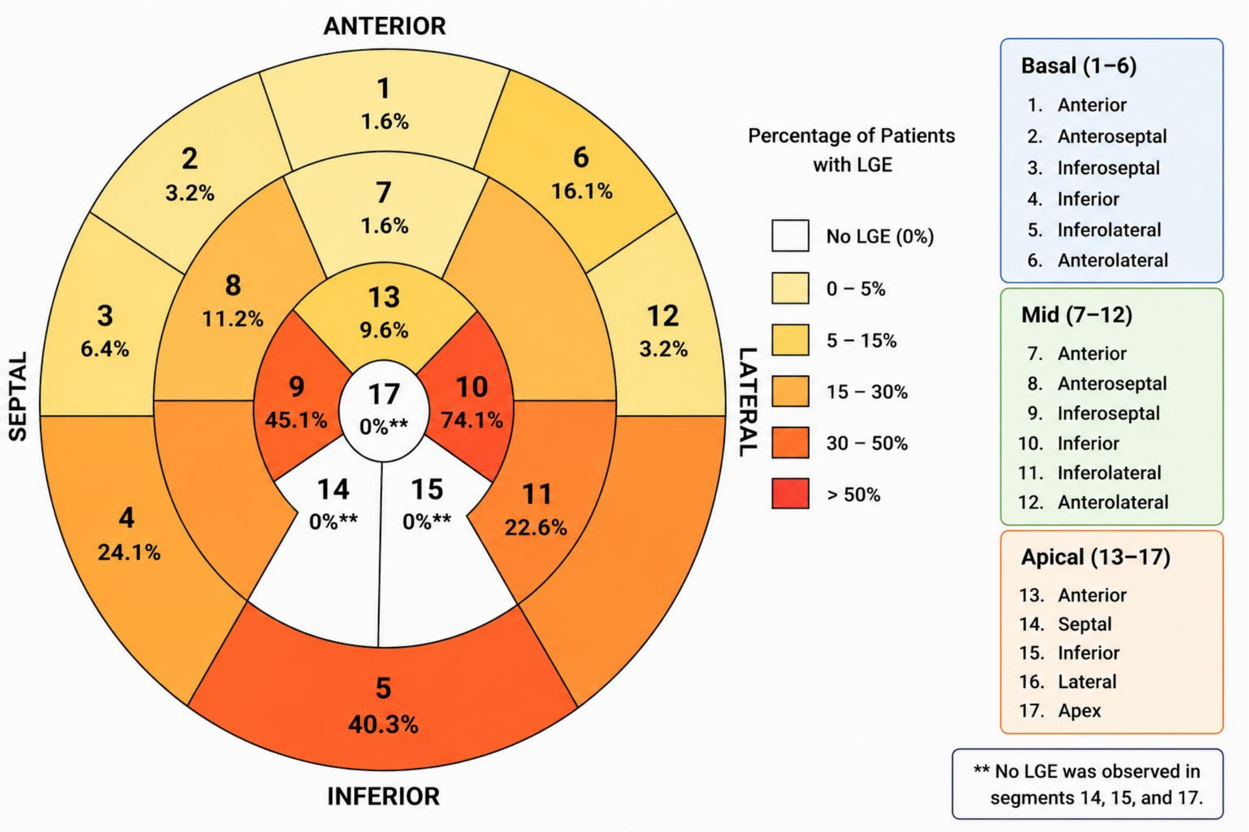


Supplementary Figure S1. Bullseye Plot of LGE Distribution Across Myocardial Segments

Supplementary Table S4 .Bootstrap Internal Validation and Calibration Analysis of the Multivariable Logistic Regression Model for LGE Prediction

| **Validation Metric** | **Result** |
|---|---|
| Hosmer–Lemeshow $\chi^2$ | 32.84 |
| Degrees of freedom | 8 |
| Hosmer–Lemeshow p value | <0.001 |
| Bootstrap repetitions | 1000 |
| Bootstrap mean AUC | 0.940 |
| Bootstrap 95% CI | 0.891–0.979 |

** Internal validation was performed using bootstrap resampling with 1000 repetitions.

Supplementary Table 5. Sensitivity Analysis Including the Interval Between Symptom Onset and CMR Acquisition in the Multivariable Logistic Regression Model for Prediction of LGE Presence

| Variable | OR (95% CI) | p value |
|---|---|---|
| SIRI | 3.47 (2.26–5.85) | <0.001 |
| fQRS-T | 1.01 (0.99–1.02) | 0.455 |
| ln(hs-troponin) | 0.99 (0.69–1.39) | 0.934 |
| ln(NT-proBNP) | 1.08 (0.75–1.54) | 0.676 |
| Glucose | 1.02 (0.99–1.04) | 0.120 |
| ln(CRP) | 0.95 (0.70–1.29) | 0.729 |
| Days from symptom onset to CMR | 1.16 (1.02–1.35) | 0.035 |

**Abbreviations:**CMR: cardiovascular magnetic resonance; CRP: C-reactive protein; hs-troponin: high-sensitivity troponin; NT-proBNP: N-terminal pro-B-type natriuretic peptide; SIRI: systemic inflammation response index.

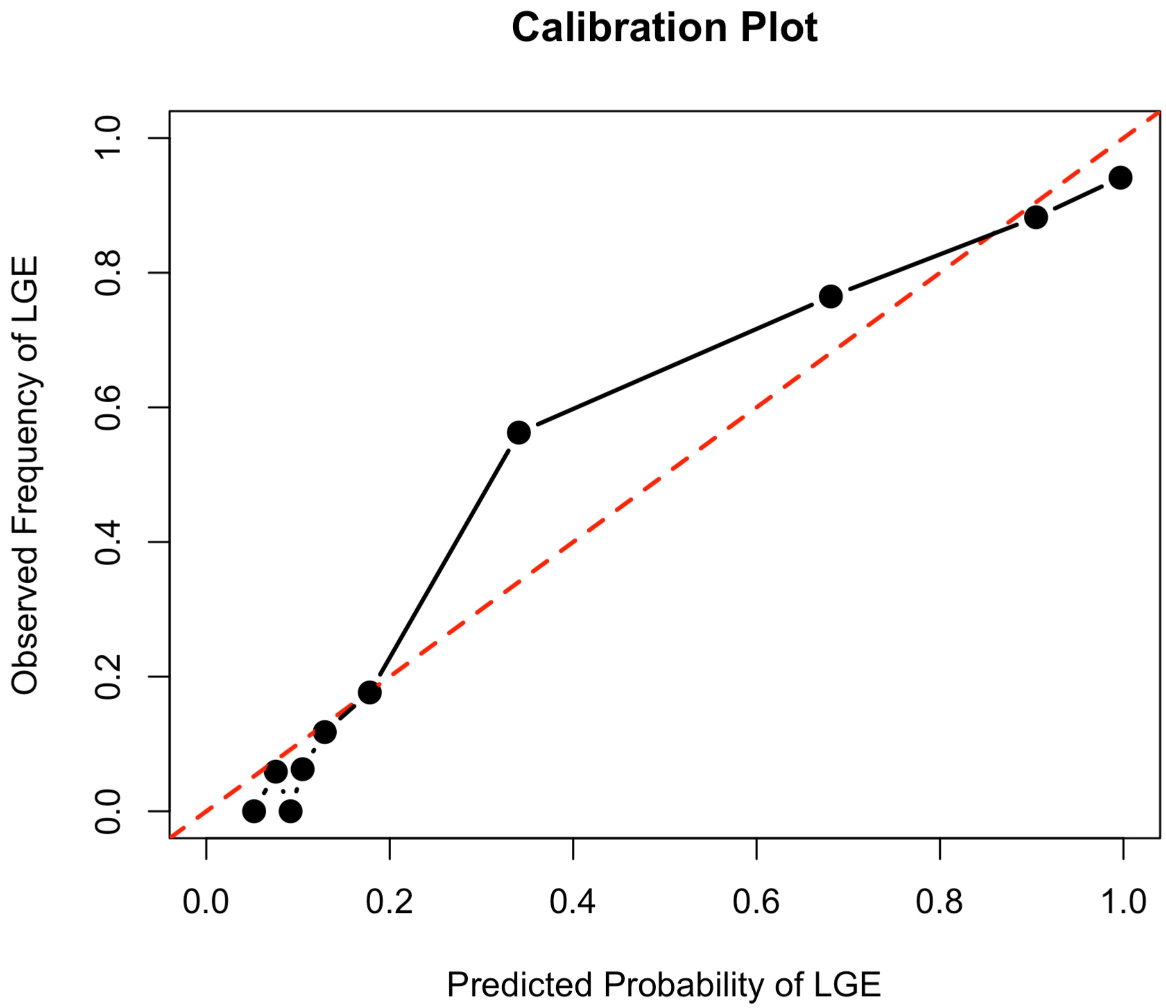


*[**]The dashed red line represents ideal calibration between predicted probabilities and observed frequencies of LGE. The black line represents observed model performance across risk groups.*

Supplementary Figure S2. **Calibration Plot of the Multivariable Logistic Regression Model for Prediction of Late Gadolinium Enhancement**
